# Pulmonary large cell neuroendocrine carcinoma with adrenal oligorecurrence successfully treated by adrenalectomy

**DOI:** 10.1002/rcr2.618

**Published:** 2020-07-14

**Authors:** Hiroki Sato, Motoko Tachihara, Tatsunori Kiriu, Masatsugu Yamamoto, Yugo Tanaka, Yoshihiro Nishimura

**Affiliations:** ^1^ Division of Respiratory Medicine, Department of Internal Medicine Kobe University Graduate School of Medicine Kobe Japan; ^2^ Division of General Thoracic Surgery Kobe University Graduate School of Medicine Kobe Japan

**Keywords:** Large cell neuroendocrine carcinoma, lung cancer, oligometastasis, oligorecurrence, surgery

## Abstract

A 63‐year‐old man suspected of having lung cancer underwent right upper lobectomy and was diagnosed with large cell neuroendocrine carcinoma (LCNEC). Eleven months after surgery, he developed an oligorecurrence in the adrenal gland and underwent left adrenalectomy. The specimen revealed LCNEC metastasis. Forty‐one months after surgery, enlargement of a lesion near the surgical site was seen. Biopsy showed LCNEC metastasis and he is currently undergoing radiotherapy for the recurrent lesion. We report a case of LCNEC with adrenal gland oligorecurrence treated by adrenalectomy, which led to long‐term survival.

## Graphical abstract

We report a case of large cell neuroendocrine carcinoma (LCNEC) with adrenal gland oligorecurrence treated by adrenalectomy, which led to long‐term survival.

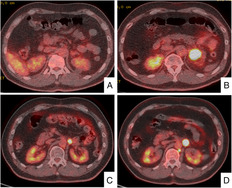

## Introduction

Large cell neuroendocrine carcinoma (LCNEC) is a relatively rare tumour that accounts for 3.1% of all primary lung cancers [1]. LCNEC is considered a non‐small cell lung cancer (NSCLC) clinically but is considered a neuroendocrine tumour according to the World Health Organization (WHO) classification. The traits are similar to those of small cell lung cancer (SCLC), and the prognosis corresponds to that of other types of NSCLC in early stages, whereas it overlaps with that of SCLC in metastatic disease [1]. Thus, LCNEC is a distinctive type of tumour within the group of NSCLCs with no consensus regarding the treatment strategy. In particular, there is no specific treatment strategy for oligometastasis or oligorecurrence in LCNEC. We report a case of LCNEC with adrenal gland oligorecurrence successfully treated by adrenalectomy, which resulted in long‐term survival.

## Case Report

**Figure 1:**
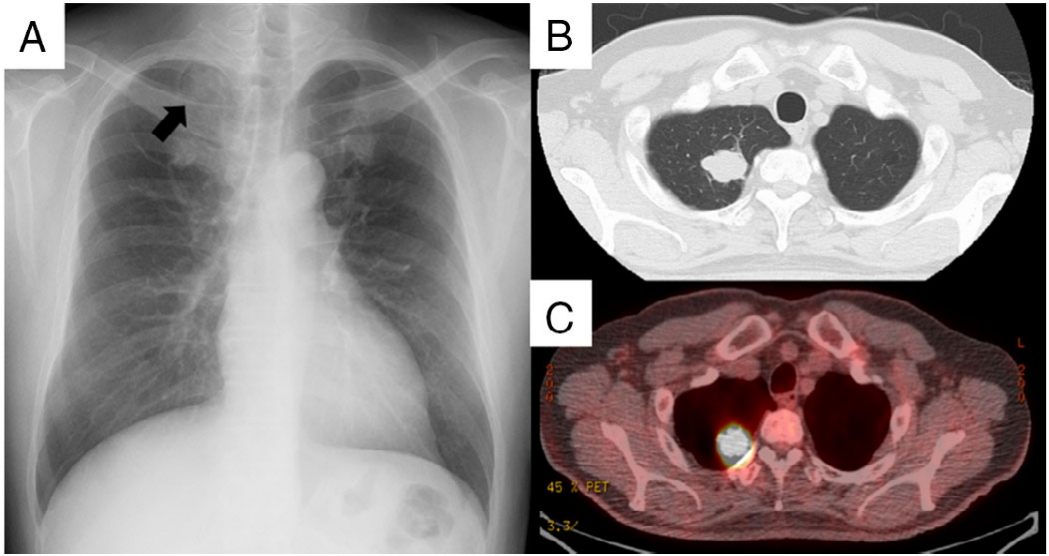
Chest X‐ray showing infiltration in the left upper lung field (arrow) at the time of referral (A). A solitary nodule with a size of 20 mm is seen on computed tomography (B). Intense uptake is shown in the lesion with 18F‐fluorodeoxyglucose positron emission tomography (C).

**Figure 2:**
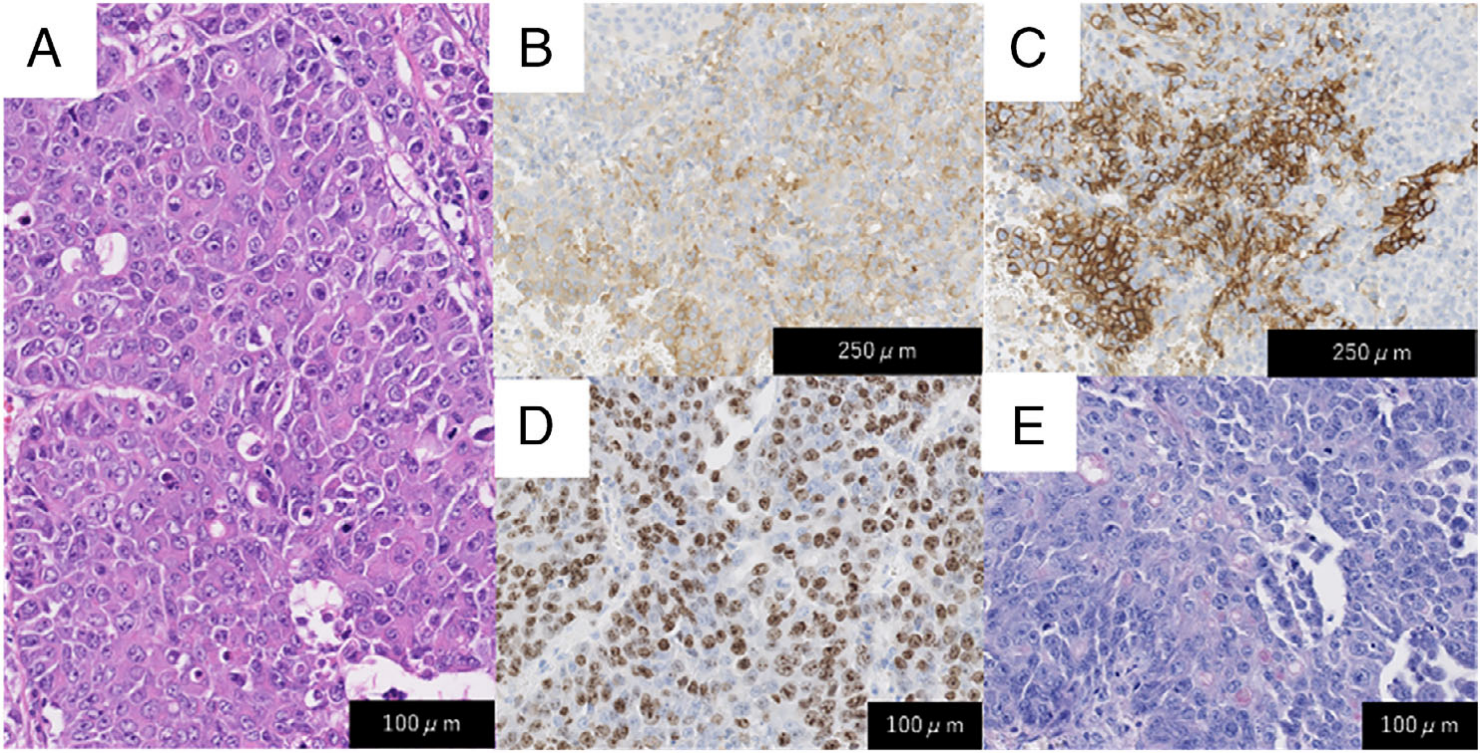
Histopathological examination of the resected specimen. Haematoxylin and eosin stain showing neuroendocrine morphology such as rosette‐like structures and peripheral palisading (A). The specimen was partially positive for synaptophysin (B) and CD56 (C). The tumour cells were 60% positive for Ki‐67 (D). Mucicarmine staining revealed intracytoplasmic mucin, but were limited to <5% of the tumour cells (E).

**Figure 3:**
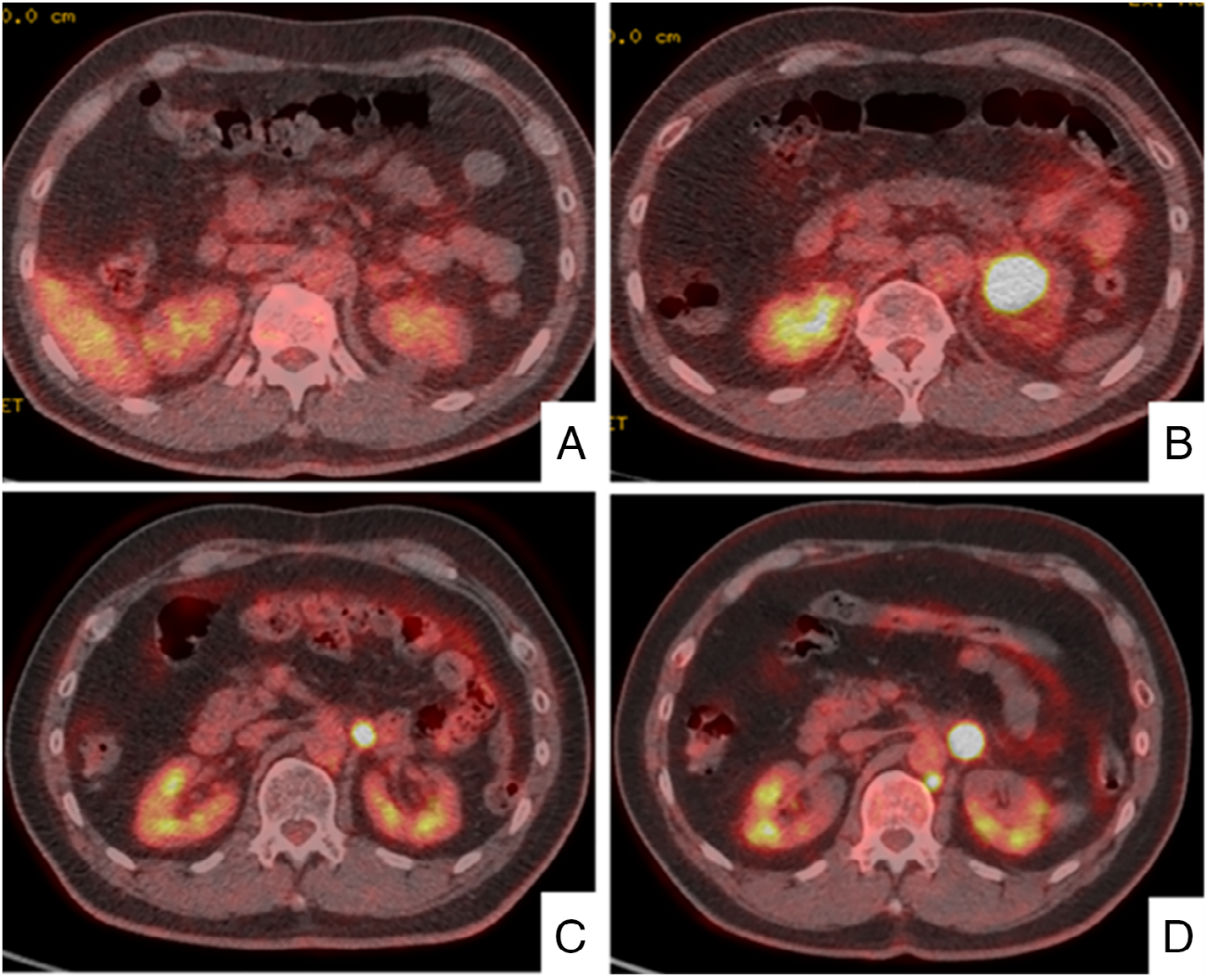
18F‐fluorodeoxyglucose positron emission tomography (18F‐FDG PET) before and after adrenalectomy. There is no sign of adrenal metastasis after thoracoscopic upper right lobectomy (A). Eleven months after surgery, an enlargement of the left adrenal gland appeared (B). Eight months after adrenalectomy, a follow‐up 18F‐FDG PET scan showed uptake in an enlarged lymph node near the resected region (C). Forty‐one months after adrenalectomy, clear enlargement of the lesion was seen (D).

A 63‐year‐old male office worker with a history of smoking 1.5 packs of cigarettes per day for 36 years was referred to our hospital due to an abnormal chest X‐ray taken for his medical check‐up. Physical examination and blood tests showed no abnormalities. A chest X‐ray showed a 20‐mm large consolidation in his right upper lung field, and 18F‐fluorodeoxyglucose positron emission tomography (18F‐FDG PET) showed an intense uptake in the same region (Fig. 1). He was suspected of having primary lung cancer, underwent bronchoscopy, and was diagnosed with NSCLC (cT1bN0M0). After diagnosis, he was referred to the thoracic surgery division where he received thoracoscopic right upper lobectomy. Histopathological examination of the resected specimen revealed neuroendocrine morphology, such as large cell size, abundant cytoplasm, rosette‐like structures, and peripheral palisading. The tumour cells were 60% positive for Ki‐67, partially positive for CD56 and synaptophysin, but were negative for chromogranin, INSM1 (insulinoma‐associated protein 1), TTF‐1 (thyroid transcription factor‐1), NapsinA, and p40. Mucicarmine staining revealed intracytoplasmic mucin, but were limited to <5% of the tumour cells (Fig. 2). Therefore, the tumour was pathologically diagnosed as LCNEC partially combined with adenocarcinoma. The clinical stage was diagnosed as pT1bN0M0. After four cycles of carboplatin and etoposide administered as adjuvant chemotherapy, he underwent periodic follow‐up computed tomography (CT) scans (Fig. 3A). Eleven months after surgery, an enlargement of the left adrenal gland appeared with no other metastatic lesions on 18F‐FDG PET (Fig. 3B). He was suspected of having solitary recurrence and underwent left adrenalectomy. The surgical specimen revealed to be compatible with LCNEC metastasis. Eight months after adrenalectomy, a follow‐up 18F‐FDG PET showed uptake in an enlarged lymph node near the resected region (Fig. 3C). Considering that the lesion was slowly progressing and no other metastasis was apparent, the decision of close observation rather than local therapy was made. There was no sign of ectopic hormone production during the course of his illness. During follow‐up, there was no change in size until 25 months after adrenalectomy, when the lesion began to shrink in size. However, 41 months after surgery, an enlargement of the lesion was shown on CT and 18F‐FDG PET scans (Fig. 3D). Endoscopic ultrasound‐guided fine‐needle aspiration was performed with histopathological examination confirming LCNEC metastasis. He underwent radiotherapy with 50 Gy/25 Fr as local therapy for the recurrent lesion.

## Discussion

LCNEC with distant metastasis is known to have a poor response to systemic chemotherapy, with a disappointingly short median survival of four months. Common sites of metastasis are the liver (47%), bone (32%), brain (23%), and adrenal gland (19%) [1].

LCNEC is a relatively rare type of lung cancer that makes randomized clinical trials difficult to perform, and the optimal therapeutic management is not well established. In general practice, surgical resection is performed for all non‐metastatic stages, which is similar to the treatment of NSCLC. However, adjuvant chemotherapy is more commonly administered, similar to the treatment of SCLC showing better survival even in stage I disease [2]. When administering chemotherapy, regimens containing cisplatin or carboplatin and etoposide are commonly chosen.

Oligometastasis has been defined as a state of cancer with a maximum of five metastases and three organs. Although these types of recurrence have been described as oligorecurrence, in most studies concerning the adrenal gland, there has been no discrimination between oligometastasis and oligorecurrence. It is considered a stage of disease between local and widespread systemic disease, in which curative treatment may be performed.

Local therapy for oligometastasis is often administered in the form of surgery or radiotherapy [3]. Several studies have shown the efficacy of adrenalectomy in NSCLC patients who have oligometastatic disease in the adrenal gland. One study consisting of 37 NSCLC patients that compared the survival of patients who underwent adrenalectomy with that of patients treated non‐operatively resulted in a better median time of survival (19 vs. six months) and five‐year survival rate (34% vs. 0%). Prognostic factors related to a better prognosis in these patients have been reported, such as presence of a metastatic lesion at the time of diagnosis and disease located ipsilateral to its origin [4].

In terms of LCNEC, there has been only one published case report in Japan, where the patient had no one‐year recurrence after adrenalectomy [5]. This is the first report in the English literature of LCNEC with adrenal oligorecurrence successfully treated by surgery showing long‐term survival.

Although the prognosis of LCNEC is generally poor, surgical resection may be an option aiming for long‐term survival in the case of oligorecurrence in the adrenal gland. It is necessary to accumulate cases and evaluate the efficacy of local therapy in LCNEC patients with oligorecurrence.

### Disclosure Statement

Appropriate written informed consent was obtained for publication of this case report and accompanying images.
